# Real-Time Prediction of Rheological Properties of Invert Emulsion Mud Using Adaptive Neuro-Fuzzy Inference System

**DOI:** 10.3390/s20061669

**Published:** 2020-03-17

**Authors:** Ahmed Alsabaa, Hany Gamal, Salaheldin Elkatatny, Abdulazeez Abdulraheem

**Affiliations:** College of Petroleum Engineering and Geosciences, King Fahd University of Petroleum & Minerals, Dhahran 31261, Saudi Arabia; ahmed.alsabaa@kfupm.edu.sa (A.A.); g201706870@kfupm.edu.sa (H.G.); aazeez@kfupm.edu.sa (A.A.)

**Keywords:** mud rheological properties, invert emulsion mud, real-time prediction, artificial intelligence, adaptive neuro-fuzzy inference system

## Abstract

Tracking the rheological properties of the drilling fluid is a key factor for the success of the drilling operation. The main objective of this paper is to relate the most frequent mud measurements (every 15 to 20 min) as mud weight (MWT) and Marsh funnel viscosity (MFV) to the less frequent mud rheological measurements (twice a day) as plastic viscosity (PV), yield point (YP), behavior index (n), and apparent viscosity (AV) for fully automating the process of retrieving rheological properties. The adaptive neuro-fuzzy inference system (ANFIS) was used to develop new models to determine the mud rheological properties using real field measurements of 741 data points. The data were collected from 99 different wells during drilling operations of 12 ¼ inches section. The ANFIS clustering technique was optimized by using training to a testing ratio of 80% to 20% as 591 data points for training and 150 points, cluster radius value of 0.1, and 200 epochs. The results of the prediction models showed a correlation coefficient (R) that exceeded 0.9 between the actual and predicted values with an average absolute percentage error (AAPE) below 5.7% for the training and testing data sets. ANFIS models will help to track in real-time the rheological properties for invert emulsion mud that allows better control for the drilling operation problems.

## 1. Introduction

During the drilling operations, drilling fluids are used to provide many functions. The primary function of the drilling fluid is to control the formation pressure of the drilled zone [1]. In addition, the drilling fluid is used to lubricate the drill bit and the whole drill string, carry the drilled cuttings up to the surface and format a filter cake to prevent further mud filtration after the filter cake creation and consequently stabilize wellbore wall in open hole section, and other functions. The commonly used drilling fluid can be classified into water-based or oil-based according to the based fluid [1,2,3]. In addition, chemical additives are added to the drilling fluid composition to adjust the fluid rheological and filtration properties in terms of the plastic viscosity (PV), yield point (YP), gel strength, and the filtrate invasion into the formation [4], in addition to the regulation of pH value, density, and water phase activity. 

The oil-based mud (OBM) is a type of the drilling fluid, and it is mainly composed of oil as a continuous phase with water content ratio less than 5%. The term “invert emulsion” is often used to represent water in oil emulsion in the oil-based mud system having water in its composition as an added component to provide a desired property [5]. The application of the invert emulsion mud is to solve the drilling problems as unstable shale, prevent damage to water-bearing formations, and, for the completion programs, as it protects the casing and tubing from corrosion [6,7,8].

There are two types for the oil-based mud systems, which are the invert emulsion and the all-oil mud types. The all-oil mud is generally designed to be free of water, but in some cases, there is a small water addition and the oil to be the external phase. When the concentration of the water addition is moderate to high (about 60%), then the system is an invert emulsion mud [9]. The invert emulsion mud system has a low toxicity effect, while the brine addition is used to control the system salinity and keep the molecules of the water within the system structure not to invade the formation [10]. The field application of the invert emulsion mud is mainly to drill the high-pressure high-temperature (HPHT) wells. That is because of its thermal stability performance that is better than the water-based mud (WBM). The invert mud system can be used in the drilling operations under temperatures up to 400 °F [11].

### 1.1. Mud Properties Measurements

The mud properties are commonly measured by conducting experimental tests. Mud balance, Fann 35 viscometer, API filter press at normal conditions and at HPHT conditions, and Marsh funnel are lab instruments used to determine the mud properties. MWT has special importance regarding the pressure control within the well [10]; therefore, it is measured (3 to 4) times/hour during the drilling operation using simple mud balance. Marsh funnel viscosity (MFV) is also called, Marsh funnel time because it is a measure of the time required for 930 cm^3^ of mud to be drained from a funnel with certain dimensions and was described by Marsh [12]. MFV is a measurement of the overall viscosity of mud in terms of time. Mud density and MFV, both are widely acceptable measurements by the drilling industry. Mud density and MFV provide an indication about the mud condition and how the mud interacts with the wellbore, and that was obtained by having samples from (in and out) mud of the well, and they are both reported with the same frequency. Precise mud rheological measurements are needed, but because they are relatively complicated compared with the mud weight and MFV, the mud rheological properties are just measured by the mud engineer (2–3) times/day. Marsh Funnel is a very simple tool and easy to use that gives a quick indication about the major general changes in the viscosity of the drilling fluid. Mud engineers and drilling crews consider the MFV changes as an indicator for the changes in mud gel strength, yield point (YP) and plastic viscosity, but they do not use MFV for prescribing solutions for mudflow problems [13]. 

The new technology application in the drilling fluid monitoring tools enabled us to have the mud properties measurements automatically [14,15,16]. Automated Marsh funnel is a new automated apparatus that can measure the Marsh funnel time and density of the drilling fluids with automated way faster than the conventional method [14]. The old fashion method was widely used, accepted, and still valid, with ongoing developments by companies and researchers to have instruments that measure the rheological properties online from flowing mud [17]. Always, there is a difference between the online and offline classic method of measuring the rheological properties due to several reasons related to the nature of mud and the reality of that mud is usually a pseudo-plastic fluid [18]. The developed models in this paper are solving this complication by describing mud rheological properties continuously or at least in higher frequency, simulating the results of the widely accepted measuring viscometers. 

### 1.2. Applications of Artificial Intelligence in the Petroleum Industry

The petroleum industry deals with a huge database within the exploration, reservoir engineering, drilling, and production operations. The application of artificial intelligence (AI) helped to deal with such big data with a learning manner to provide the internal relations between the inputs and the outputs. AI is an approach that appeared to overcome the weaknesses in the analytical models. Generally, many AI applications in the petroleum industry have been developed [19,20]. There are five AI techniques, which are artificial neural networks (ANN), support vector machines (SVM), fuzzy inference systems, neuro-fuzzy, and ensemble models [21].

Lim and Kim [22] used the fuzzy logic technique to estimate the reservoir porosity and permeability from the well logs. Another study was performed to predict the reservoir porosity from the wireline log data using AI tools as ANN, ANFIS, and SVM with a good result [23]. A self-adaptive differential evolution integrated with artificial neural network (SaDE-ANN) was used to predict the reservoir permeability from the well logs [24]. Mahmoud et al. [25] introduced a newly developed correlation to determine the static Young’s modulus from the log data using the clustering technique. Elkatatny et al. [26] studied the sonic travel time prediction from the well log data using the ANN, ANFIS, and SVM tools.

In drilling engineering, many studies were performed to apply AI science for real-time optimization of the drilling parameters [27], ANN was used for the optimization of the drilling rate of penetration using 3333 actual data points [28]. Al-AbdulJabbar et al. [29] introduced a study for rate of penetration (ROP) optimization using AI by considering the effect of drilling and mud properties on the ROP performance.

In addition, the AI application was used for the reservoir rock geomechanical properties prediction. Tariq et al. [30] presented a study for unconfined compressive strength (UCS) prediction using the ANN, ANFIS, and SVM tools from the well logs. Another study predicted the rock failure parameters from the well log data using the ANN, ANFIS, and SVM tools [31]. ANN was applied to predict the rock UCS for the carbonate reservoir [32]. Additional rigorous empirical correlations based on the weights and biases of ANN to predict sonic times, rock elastic parameters, and UCS was performed and tested with real field data with high accuracy performance [33]. Elkatatny [34] provided an ANN prediction model to determine the rock static Poisson’s ratio using the wireline logs data. ANN, ANFIS, and SVM tools were used to estimate the P-wave and S-wave travel times from the well logs data with a low error less than 5% AAPE; the results showed that ANN outperformed the ANFIS and SVM results [35]. Elkatatny et al. [36] developed an accurate and robust correlation for static Young’s modulus estimation from log data without the need for core measurements. Therefore, many AI applications succeeded to provide a geomechanical properties prediction from the well logs with high accuracy without the core analysis time and cost. And additional work for predicting the drilling fluids rheological properties was performed for many mud types due to the importance of the drilling fluid properties optimization and monitoring in the drilling operations [37]. 

It was believed that the rheological properties of mud are related by somehow to the MWT and MFV. However, the trials to find the relation by conventional mathematical methods [38,39] did not provide the required accuracy. Pitt tried to find a mathematical correlation to relate MWT measured in (g/cm^3^) and Marsh funnel viscosity (MFV) measured in (seconds) to the apparent viscosity (AV) measured in (cP) via Equation (1). The constant in Pitt correlation was modified by Almahdawi et al. [38] from 25 to 28 as per Equation (2).

AV = MWT × (MFV − 25)(1)

AV = MWT × (MFV − 28)(2)

Predicting the drilling fluid rheological properties with time using the new artificial intelligence techniques helps to optimize the best performance for the drilling fluid and prevent the drilling problems [4]. Razi et al. [40] used the feedforward multilayer perceptron (FFMLP) neural network to predict the WBM rheology, while ANN was used as a prediction model for the WBM [41]. Elkatatny et al. [42] introduced for the first time a new prediction model for the drilling fluid rheological properties from the Marsh funnel viscosity, solid content, and density measurements and the model used the ANN technique and provides a mathematical model from the weights, biases, and the transfer function within the ANN architecture. Another mud type was studied for the application of ANN to predict the KCl-polymer mud rheological properties [43]. The study used the mud density, Marsh funnel viscosity, and solid percent as inputs to predict the rheological properties (plastic viscosity, apparent viscosity, yield point, flow behavior index, and consistency index of the drilling fluid. Da Silva Bispo et al. [44] provided a study to estimate the apparent viscosity of WBM using a feedforward multilayer perceptron (FFMLP). The model was optimized by six neurons in the hidden layer and using hyperbolic activation functions in the hidden layer and linear activation function in the output neuron. ANN tools were applied to predict the rheology of oil-based mud system by Al-Azani et al. [45] and the study predicted the rheological properties such as the plastic viscosity, apparent viscosity, the rheometer readings at 600 and 300 revolution per minute (rpm) and the flow behavior index for oil-based mud from the mud weight, the Marsh funnel viscosity and solid content and the results showed that the correlation coefficient was higher than 90%. Elzenary et al. [46] studied the prediction of the equivalent circulating mud density while drilling using two AI tools, which are artificial neural network (ANN) and adaptive neuro-fuzzy inference system (ANFIS). Hoang [47] used Fuzzy logic model, feedforward artificial neural network model (ANN), feedback artificial neural network model, and support vector regression (SVR) to predict the viscosity of the non-Newtonian drilling fluids. 

Recently, Gowida et al. [48] used the ANN to predict the CaCl_2_ brine-based drill-in fluid properties. Elkatatny et al. [37] presented a new approach to predict the mud rheology of NaCl water-based drill-in fluid using AI to provide five correlations for the rheological properties and three input parameters which are mud weight, Marsh funnel viscosity and solid volume percent. The models showed a coefficient of determination (R^2^) higher than 90% between the field and the calculated values. It is clear that a lot of work was performed to predict the rheological properties of different drilling fluids types with the help of AI tools applications. 

From an economic point of view, the drilling fluids cost share 25–40% of the total well drilling cost [49]. Therefore, designing and monitoring the drilling fluids parameters are very critical for drilling operations. A bad mud design or non-precise monitoring for the drilling fluid will cause drilling problems, and therefore, add an extra cost to the drilling expenditures [50,51]. The mud rheology optimization is required as it affects the rate of penetration (ROP) and rig hydraulics [52,53]. Consequently, monitoring the drilling fluid in real-time will help to complete the drilling operation in accordance with a successful technical and economic program.

As per of that importance of the mud properties design and monitoring during drilling, this paper aims to utilize ANFIS optimization tool to build models that can predict the invert emulsion mud rheological properties as plastic viscosity, (PV), yield point, (YP), behavior index (n), which indicates the degree of the fluid shear thinning as the less n value, the greater the shear-thinning characteristic, viscometer reading at 300 (R_300_), viscometer reading at 600 (R_600_), and apparent viscosity (AV), depending only on MWT and MFV. The study presented in this paper added a contribution to the efforts of fully automating the process of retrieving rheological properties with high accuracy and over a real-time for invert emulsion mud system. For the first time, this study provides a new technique to predict the rheological properties of invert emulsion mud in real-time from only two inputs MWT and MFV. 

### 1.3. Adaptive Neuro-Fuzzy Inference System (ANFIS) 

The target of this study was to build an initial Sugeno-type FIS using subtractive clustering technique to be used in ANFIS training of the data set. ANFIS is an adaptive neuro-fuzzy inference system (ANFIS), which is a type of artificial neural network (ANN) that is depending on the Takagi–Sugeno fuzzy inference system. ANFIS technique has been developed in the early 1990s. Its inference system is using a set of fuzzy if-then rules that have learning capability to optimize functions. ANFIS provides the best advantages that exist in both fuzzy logic and neural network techniques in one tool [54,55]. The ANFIS uses the backpropagation (BP) algorithm and the least square to get the best membership function that uses for training the inputs and outputs data sets [56].

The rest of the paper is structured as follows, Section 2 contains details about the data and the methods used to develop the models and the used approaches. Section 3 showed the obtained results from the models. The discussion is presented in Section 4 about the main findings from this study and a comparison of the results with the previous work. The conclusion section discusses the meaning and application of this study.

## 2. Materials and Methods

The drive for building this ANFIS model was to exclude the cumulative errors from manual conventional measurements and reporting of mud properties in drilling operations, as described later. The second main drive is to have a higher resolution for mud rheology. The developed ANFIS model can be a part of an expert system that automatically obtains MWT and MFV and rheological properties every 15 to 20 min with minimal errors. The approach that was finally chosen for developing the ANFIS models was depending on measurements database of mud properties from the daily mud reports recorded for invert emulsion mud throughout a duration of almost one year of drilling operations within a province in the Middle East. As explained before, the measuring and reporting processes are all manual, and human errors may cause some wrong, misleading data to be recorded.

The data was collected from real field measurements performed by mud engineers on the rig sites of 99 wells. Invert emulsion mud was preferred to be used during drilling the 12 ¼ inches section for all of the wells from which the mud measurements were collected. All of the measurements were for the invert-emulsion mud type only but for different formulations through the 12 ¼ inches section.

Seven hundred forty-one points were aggregated after preprocessing and data cleaning to be used as a feed to the ANFIS model with training to testing ratio 80% to 20%. The collected data were representing the mud weight measured using a mud balance in pounds per cubic foot (pcf) and Marsh funnel viscosity (MFV) measured using Marsh funnel and represented in seconds. In addition to MWT and MFV, the mud rheological properties are measured using a rheometer, which is usually a rotational type rheometer. The rheology tests were performed at atmospheric pressure according to recommended practice for field testing oil-based drilling fluids by API RP 13B-2 [57]. The simple measurements of MWT and MFV are measured frequently 2–4 times per hour during the drilling operation due to their simplicity. Instead of MFV, other rheological properties are used for a better understanding of mud problems and corrective actions. The rotational rheometers are used to measure the shear stress in (lb/100 ft^2^) under certain shear rate applied by a rotational speed in (rpm) given to the outer cylinder of two co-axial cylinders between, which the mud is existing. Shear rate and shear stress data are used to calculate the mud plastic viscosity (PV) and yield point (YP) for the mud rheological properties. The data used in this paper was containing PV, and YP values along with mud weight and Marsh funnel time for the same samples. The following relations were used to determine the PV, YP, and n values from the shear stress readings at 300 rpm and 600 rpm, which are (R300 and R600) [3]:

(3) $$\mathrm{PV}=R_{600}-R_{300}$$

(4) $$\mathrm{YP}=2{\;R}_{300}-R_{600}$$

(5) $$\mathrm{AV}=\frac{R_{600}}{2}$$

(6) $$n=3.32\times \log\left(\frac{R600}{R300}\right)$$

**Figure 1:**
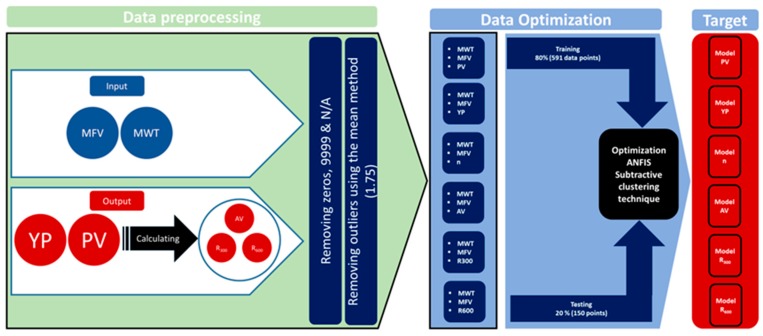
Flow chart for developing the adaptive neuro-fuzzy inference system (ANFIS) models.

The flow chart in Figure 1 is showing the details about the preprocessing of the data and the optimization method to reach the target at the end. The preprocessing included first the removal of all zeros, N/A’s and missed data in addition to the illogic values depending on experience and engineering since before using a code to automatically remove all rows of outliers, depending on the mean of the data with a threshold value of 1.75 to assure the quality of the training process.

### 2.1. Data Statistics

Real field mud samples were used in this study after preprocessing from the outliers, invalid wrong readings, and repetitions to be 741 points that were used to build the AI model. The model was trained and validated using the ANFIS. Data descriptive statistics in Table 1 show the statistical analysis for the input and output parameters.

**Figure 2:**
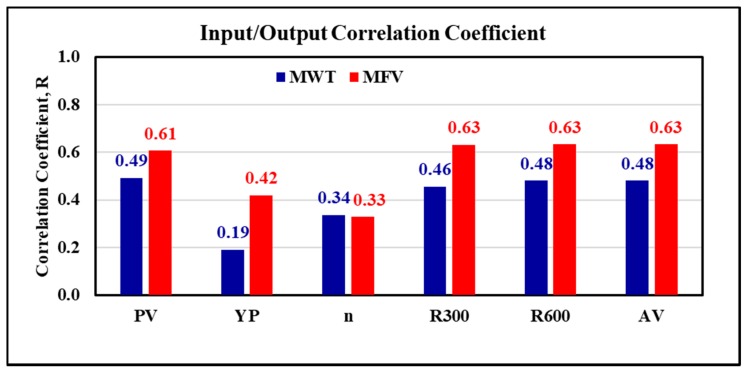
The correlation coefficient between the inputs and outputs.

The data showed that the minimum value for the mud weight is 67 pcf, and the maximum value was 98 pcf. The MFV rage from 45 to 98 s, while the PV showed a minimum value of 13 cP, and the maximum PV is 47 cP. The yield point for the mud data had a range of 10 to 31 lb/100 ft^2^. The AV showed a range of 20 to 59.5 cP. It is clear that the collected data is representing a wide range of readings that commonly found in the drilling operations using invert emulsion mud type for 12 1/4 inches section. The correlation coefficients between the inputs and outputs were calculated. R between MWT and MFV, which are representing inputs data from one side, and all other values of outputs (PV, YP, n, R_300_, and R_600_) from the other side, show a strong direct relationship with most of the outputs as it appears from Figure 2. It is clear that the correlation coefficient is higher than 0.46 between the MWT and all the outputs except with the YP and n, where the R shows 0.19 and 0.34 for YP and n, respectively. Where the R for the MFV and the outputs show higher values than 0.61 for all outputs except for the YP, and n at which the R shows 0.42 and 0.33, respectively.

### 2.2. Methodology and Building ANFIS Models

Several trials were performed to get the optimum training/testing ratios, cluster radius, and the number of epochs. The subtractive clustering technique was used to cluster the data and creates a fuzzy inference system with a minimum number of rules. The subclust function was used to determine the number of membership functions and rules. Several trials were made to optimize the cluster radius value that determines the cluster center range of influence on its spherical neighborhood. The radius value was determined to be 0.1 times the width of the data space. The number of epochs was affecting too much the time needed for training the model, and it was realized that more than 200 epochs did not improve the results that much; therefore, 200 epochs were chosen to train the models and also, to avoid overfitting that would happen with a large number of epochs. The optimization process was coded using Matlab on a workstation with moderate specifications to minimize the time needed for the runs. Other programming languages can be used to develop the ANFIS code but Matlab was chosen because it is easy to use and has many functions already built on it, which is time and effort-saving. To evaluate the ANFIS results, AAPE was calculated using Equation (7)

(7) $$\mathrm{Average}\;\mathrm{Absolute}\;\mathrm{Percentage}\;\mathrm{Error}\;\left(\mathrm{AAPE}\right)=(\frac{1}{N}\;\sum_{i=1}^{N}\left|\frac{\mathrm{Xi}\;\mathrm{actual}-\mathrm{Xi}\;\mathrm{calculated}\;}{\mathrm{Xi}\;\mathrm{actual}}\right|)\times 100$$

where, N is the number of data points, and Xi represents the rheological parameter 

## 3. Results

**Figure 3:**
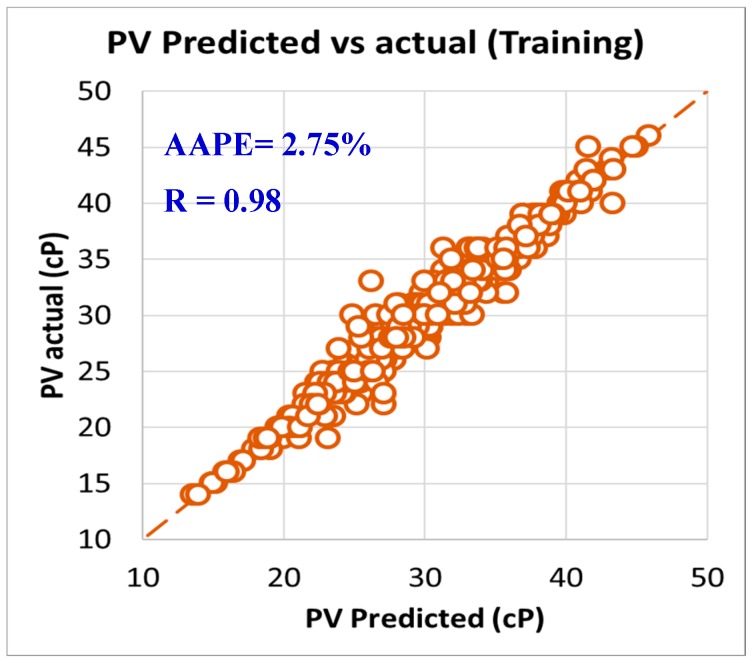
PV predicted vs. actual (training).

**Figure 4:**
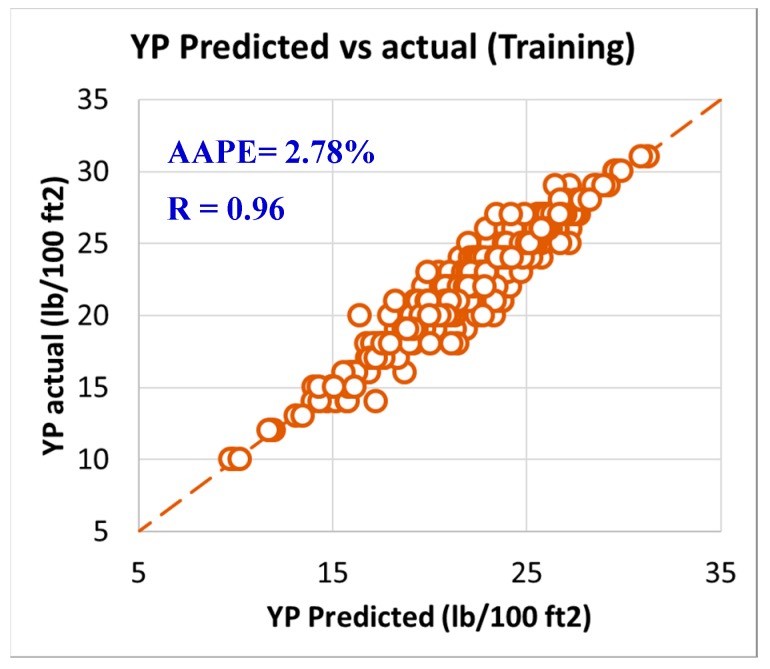
YP predicted vs. actual (training).

**Figure 5:**
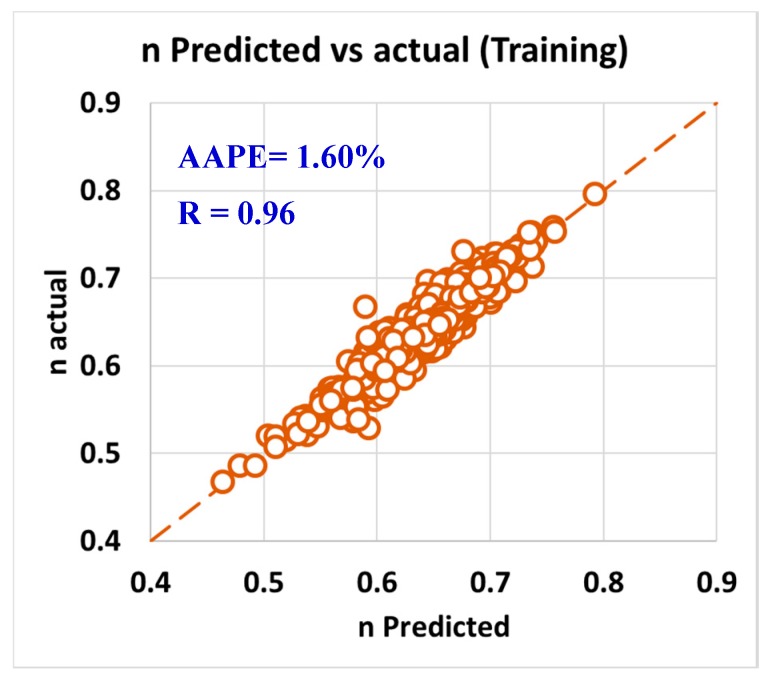
n predicted vs. actual (training).

**Figure 6:**
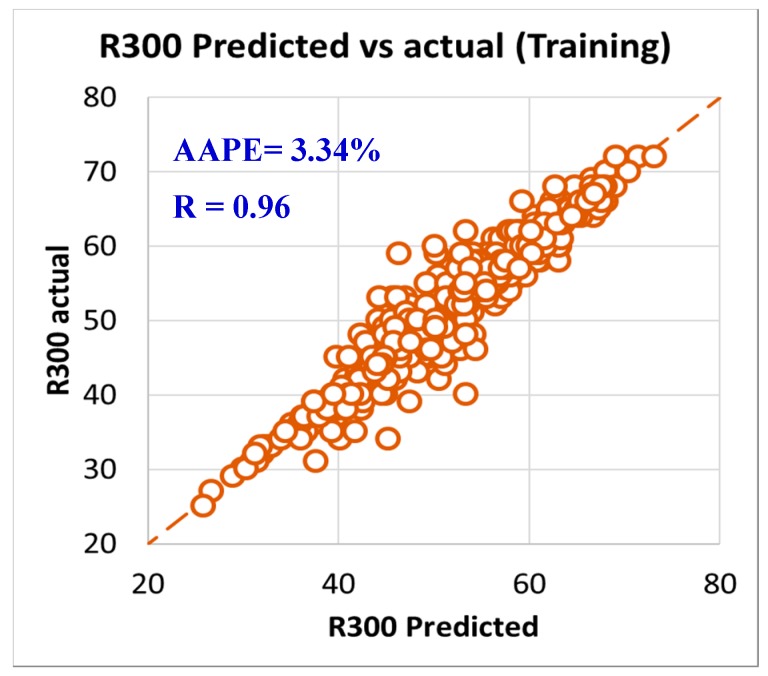
R_300_ predicted vs. actual (training).

**Figure 7:**
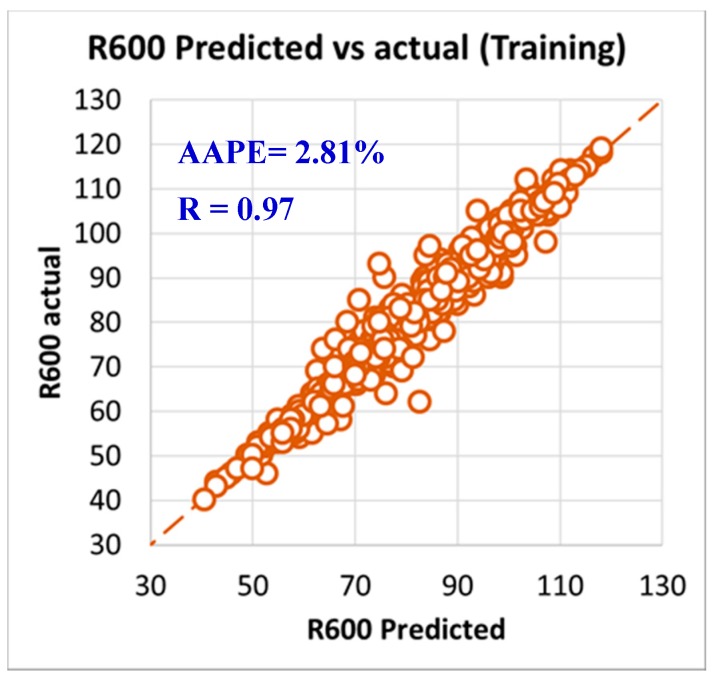
R_600_ predicted vs. actual (training).

**Figure 8:**
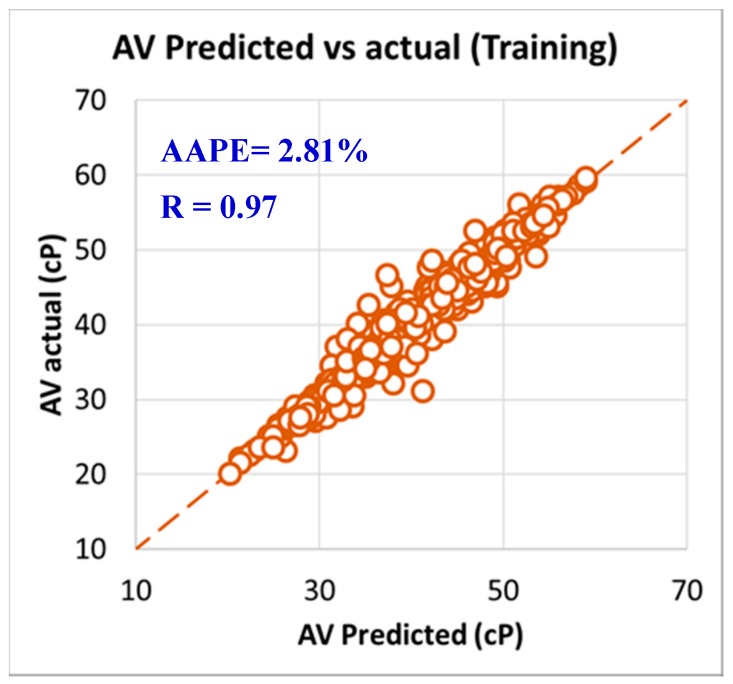
AV predicted vs. actual (training).

As per the objective of this paper to utilize the field data and measurements in developing models to predict rheology properties from the MWT and MFV, it was needed to have a training data set that is used to develop the models and training data. In order to have this optimum ratio of training to testing data set, it was decided to try different training to testing ratios starting from 50%:50% then, increasing the training ratio over the testing ratio on steps. The training/testing ratio of the data were optimized to be 80% of the data for training (591 data points) and 20% of the data for validating the models (150 points) by testing it which resulted in very accurate models for predicting the PV, YP, n, R_300_, R_600_, and AV. The correlation coefficients between the predicted and actual data for all the six models showed values higher than 0.96, while the AAPE did not exceed 3.34% for the training data set (591 data points). The accuracy of the plastic viscosity ANFIS model results of the training data set is shown in Figure 3, as the results showed a very high correlation coefficient that was 0.98, and this was the highest R among all the other developed models in this paper. R was 0.96 for the yield point, as represented in Figure 4, and AAPE was 2.78% that was almost the same as for PV model. The R for the behavior index (n) was 0.96 while the AAPE was 1.6%, as shown in Figure 5, and R was 0.96 for R_300_, and the AAPE was 3.34% as in Figure 6. For the R_600_, the correlation coefficient was 0.97 and 2.81% AAPE, as showed in Figure 7. Figure 8 represents the accuracy for the apparent viscosity model training, as R was 0.97 and the AAPE was 2.81%.

**Figure 9:**
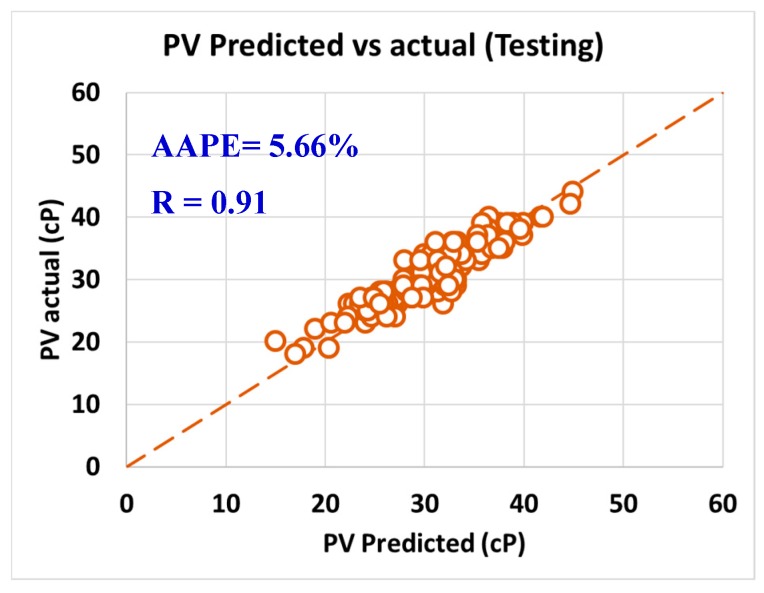
PV predicted vs. actual (testing).

**Figure 10:**
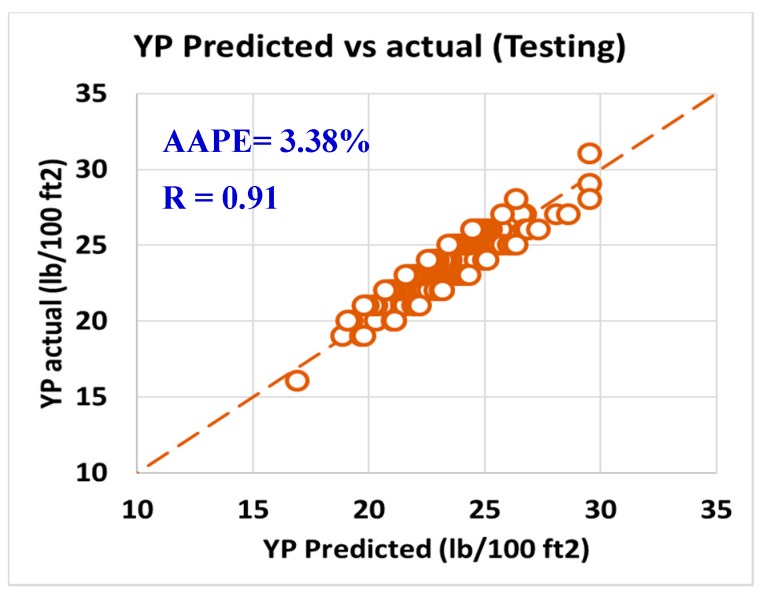
predicted vs. actual (testing).

**Figure 11:**
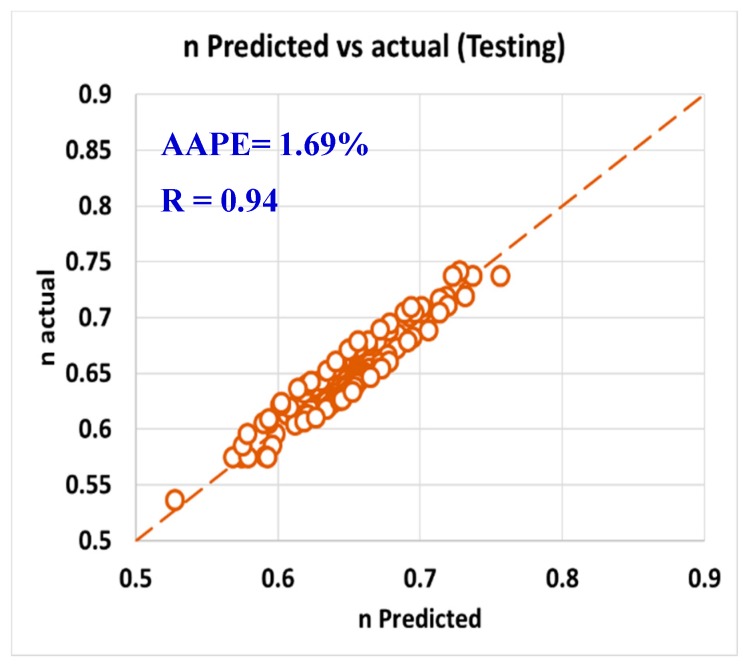
n predicted vs. actual (testing).

**Figure 12:**
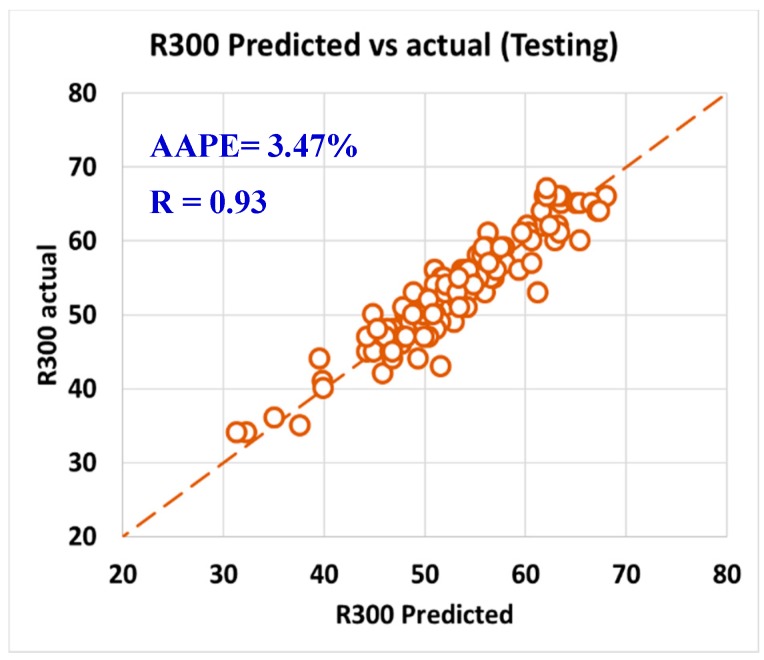
R300 predicted vs. actual (testing).

**Figure 13:**
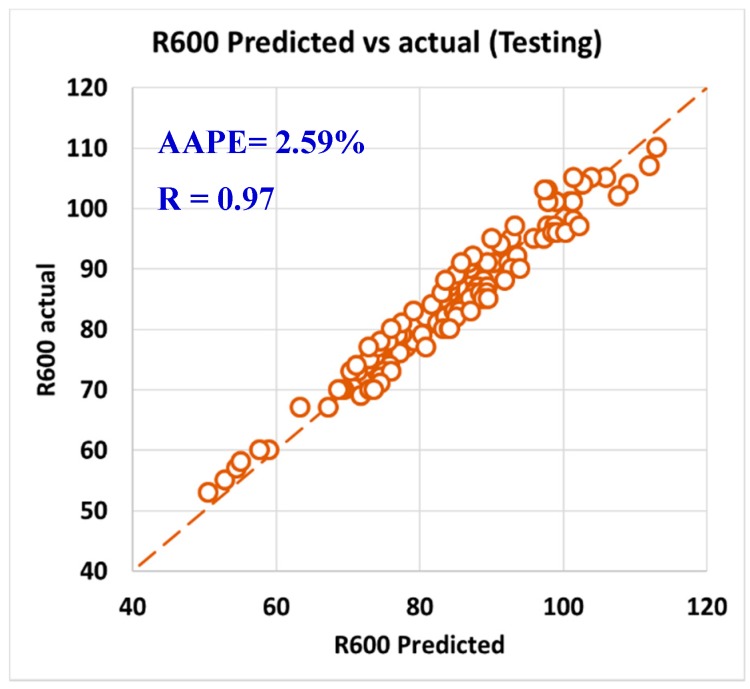
R_600_ predicted vs. actual (testing).

**Figure 14:**
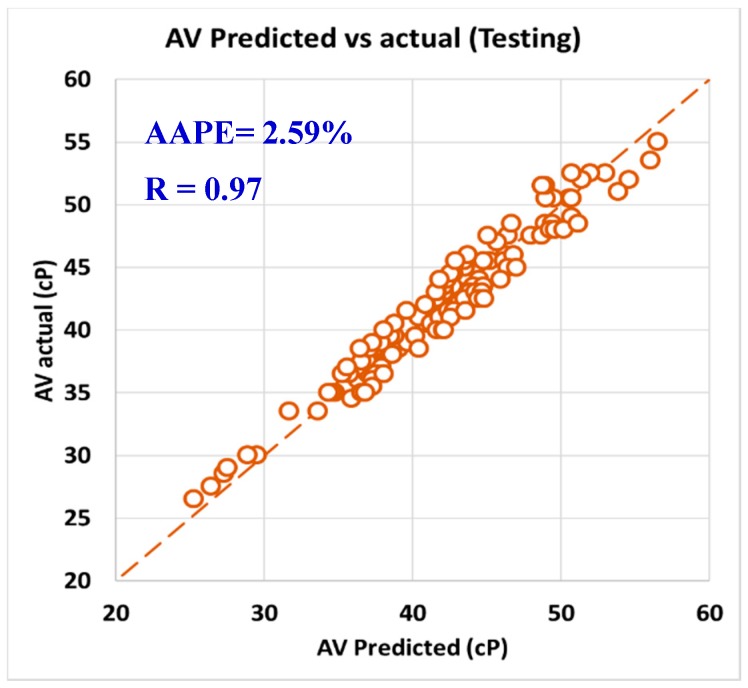
AV predicted vs. actual (testing).

For validating the six models (PV, YP, n, R_300_, R_600_, and AV), the models were tested using 20% of the data (150 rows of data measurements) and the models showed acceptable results as the correlation coefficient was about 0.91 and the AAPE did not exceed 5.66%. When testing the PV ANFIS model with 150 points, the correlation coefficient was 0.91 and AAPE was 5.66%, which a very good result as in Figure 9. Yield point model results were compared to the actual values, and R showed 0.91 and 3.38% AAPE as in Figure 10. Figure 11 represented the results accuracy for n model as R showed 0.94 for the testing data and 1.96% AAPE. R_300_ results from the developed model were shown in Figure 12 and compared to actual data, found that R was 0.93, and AAPE was 3.47%. Apparent viscosity (AV) and R_600_ had the most accurate models with R was 0.97, and AAPE was 2.59, as shown in Figure 13 for R_600_, and Figure 14 for AV results.

## 4. Discussion

The root problem of measurement of the rheology of mud is the low resolution of the measurements because of the difficulty of performing the lab tests. The viscometer used to get rheology properties is more suitable for research and lab measurements that will be in absolute units [12]. In the field, there was a need for quick estimation of viscosity with higher frequency. This was resolved by Marsh [12] as he proposed the usage of a funnel to get a rough estimation of the viscosity, which is not in absolute units of viscosity and just a comparative or relative measurement. Marsh funnel was used since that along with viscometers with still a need to have more accurate measurement like the ones we get from the viscometers but, in higher frequency like what we get from the funnel. There was a strong tendency within the industry to replace the Marsh funnel and the viscometers with automatic measurements for rheology. Pipe viscometers had popularity within the industry as a replacement. Vajaragah and Oort [17] introduced a type of pipe viscometer that is indirectly measuring the rheological properties with the possibility of having cumulative errors and deviation when compared to viscometer readings. Away from that, Pitt [38] proposed a relationship between Marsh funnel viscosity and apparent viscosity, which was later modified by Almahdawi [39], which is not confirmed to be suitable for all mud types and not validated with acceptable accuracy. Elkatatny [14] had a patent of automated Marsh funnel that can have MWT and MFV readings automatically without any human interference eliminating the errors that may exist due to the manual measurements. Having the readings of Marsh funnel automatically is opening the door to investigate more the relation between MFV, MWT, and other rheological parameters. Elkatany et al. [42] had used artificial neural networks to develop empirical models that can predict the PV, YP, n and fluid consistency with high accuracy but, he included the solid percentage in mud in his empirical correlations that again is still not benefitting from the patented automated Marsh funnel that has no sensor for measuring the solid percentage in the mud sample.

This gap is filled with this work, which introduces models for predicting rheological properties from only the MWT and MFV, which can be measured automatically by the mentioned technique of the automated Marsh funnel. A computer managing system can convert the raw readings of mud weight, and Marsh funnel viscosity into plastic viscosity, yield point, apparent viscosity, behavior index, and even viscometer reading at 300 and 600 rpm using the developed ANFIS-based models in this paper. The accuracy of the models against the validating data in the testing phase was excellent as the training range was the same range of the full data set for inputs and outputs to have the most accurate models. The testing data was covering almost the same range to assure generalization of the generated models. Continuous validation and improvement for such models generated by artificial intelligence is required to improve their accuracy. However, the amount of data used and its range used in building the models are satisfying as per the results achieved during training and validating processes. A comparison between the ranges of output datasets for training and the output datasets for testing is showing that the models were tested against almost the same ranges of the training datasets in Table 2. As shown in the table, the training dataset range covers the testing dataset range as an example of the minimum value for PV was 13 cP for the training data while it was 18 cP for the testing data set, the maximum PV value was 47 cP for training data set and 44 cP for the testing data set. 

### Predicted R_300_ and R_600_ for calculating PV and AV for Comparison with Previous Studies

In order to authenticate the quality of the work, two steps were applied to show up the reliability of the ANFIS models. The first step was that the Predicted R_300_ and R_600_ were used for calculating PV and AV, and then the results were compared to actual values. This comparison adds up to the strength of the ANFIS models. The second step was, comparing the predicted AV and the calculated AV from the predicted R_600_ and R_300_. In addition, the predicted AV was compared to the AV correlations from the literature.

**Figure 15:**
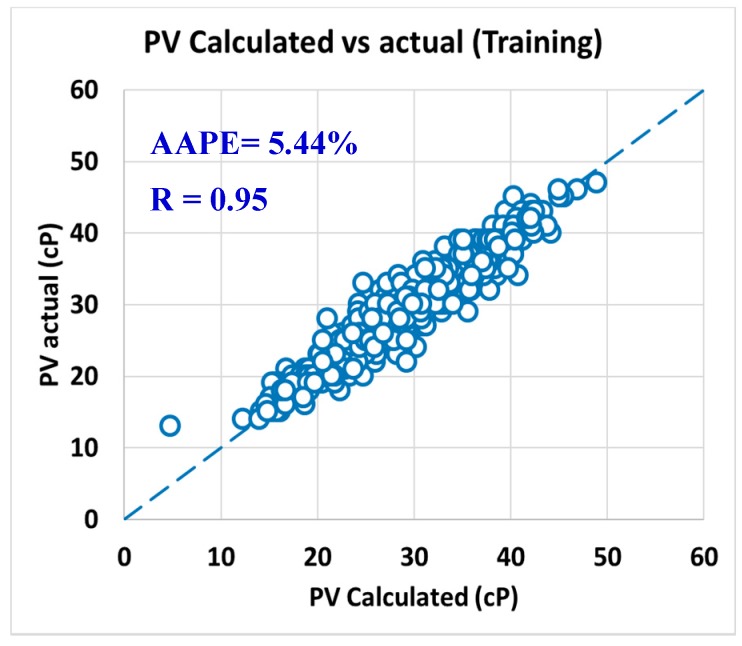
Actual PV vs. calculated PV from predicted R_300_ and R_600_ (training).

**Figure 16:**
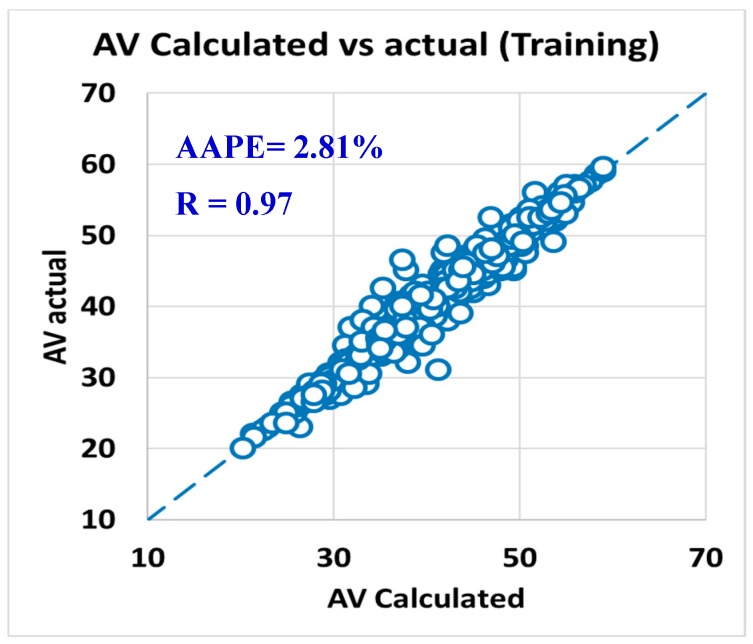
Actual AV vs. calculated AV from predicted R_600_ (training).

The predicted R_600_ and R_300_ were used to calculate the PV and AV as per Equation (1) and Equation (3). Figure 15 presented the R-value between the actual PV versus the calculated PV from the predicted dial readings at 300 and 600 rpm, where the R was 0.95, and AAPE was 5.44% for using the training data set. For AV, the R was 0.97, and the AAPE showed 2.81% as in Figure 16.

**Figure 17:**
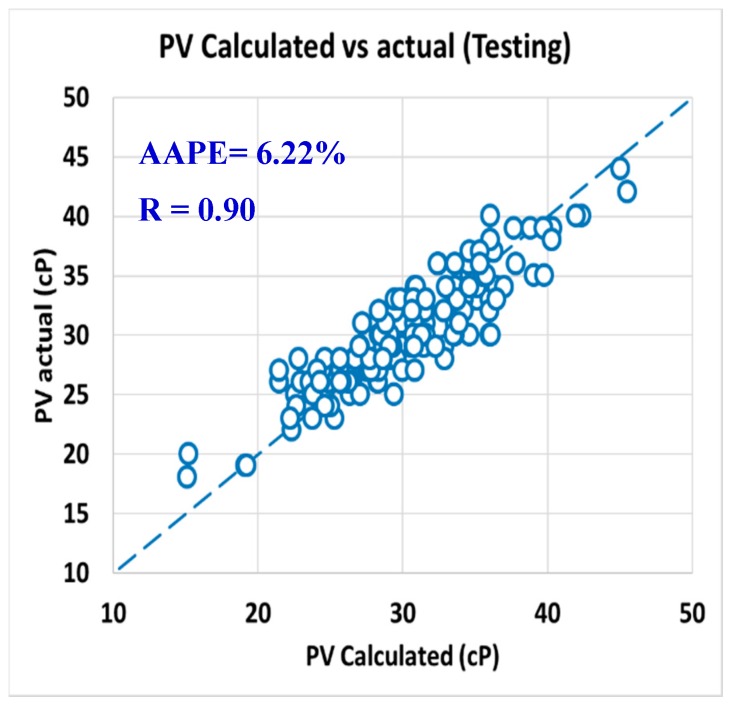
Actual PV vs. calculated PV from predicted R_300_ and R_600_ (testing).

**Figure 18:**
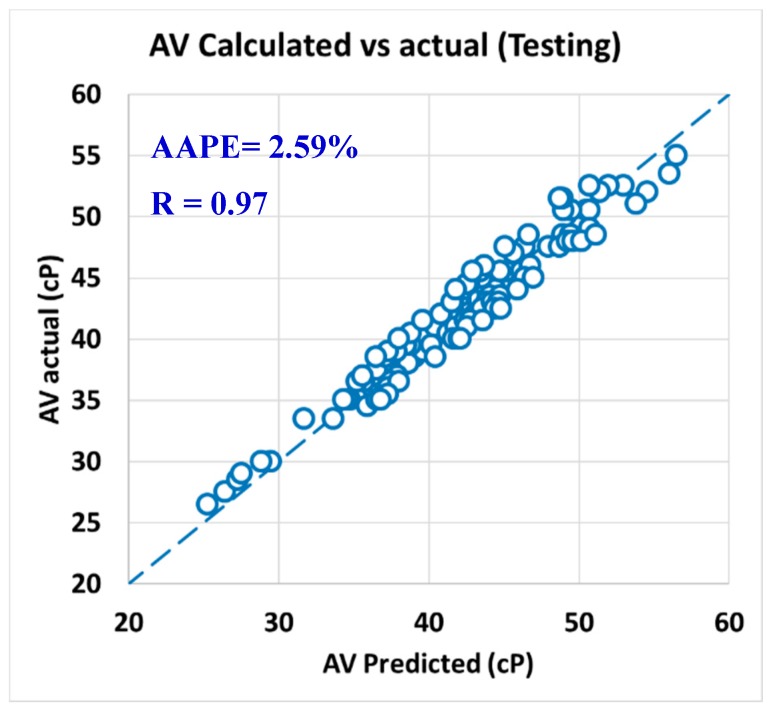
Actual AV vs. calculated AV from predicted R_600_ (testing).

For the testing data set, the correlation coefficients (R) were 0.90 and 0.97 for PV and AV, respectively, with still low AAPE as it was 6.22% for PV and 2.59% for AV, as shown in Figure 17 for PV and Figure 18 for AV. 

**Figure 19:**
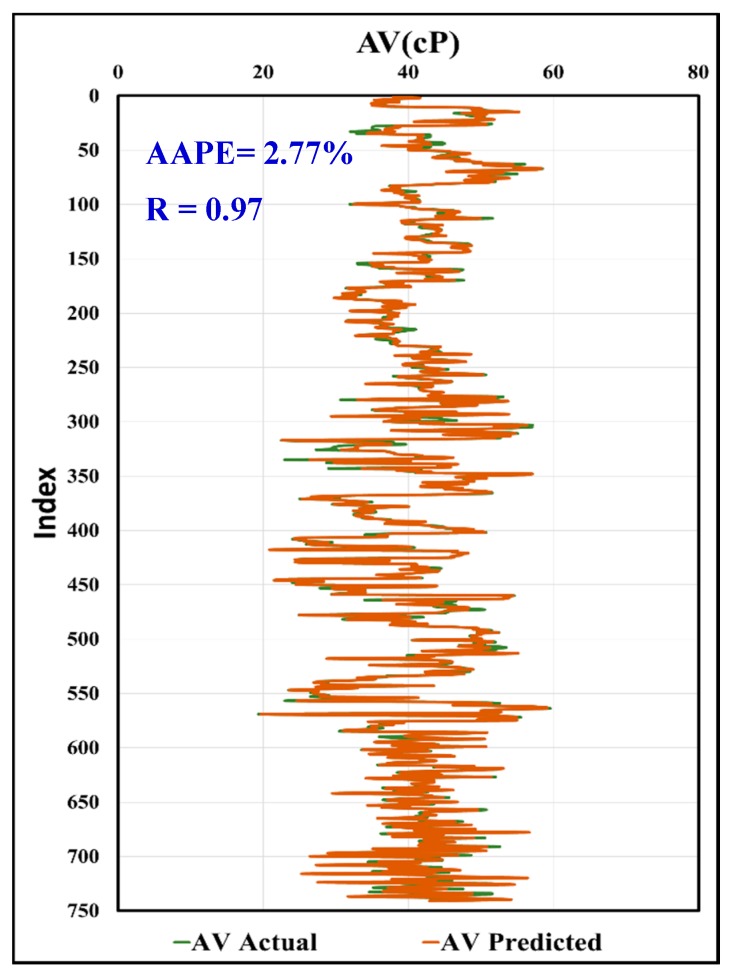
Predicted AV vs. actual values.

**Figure 20:**
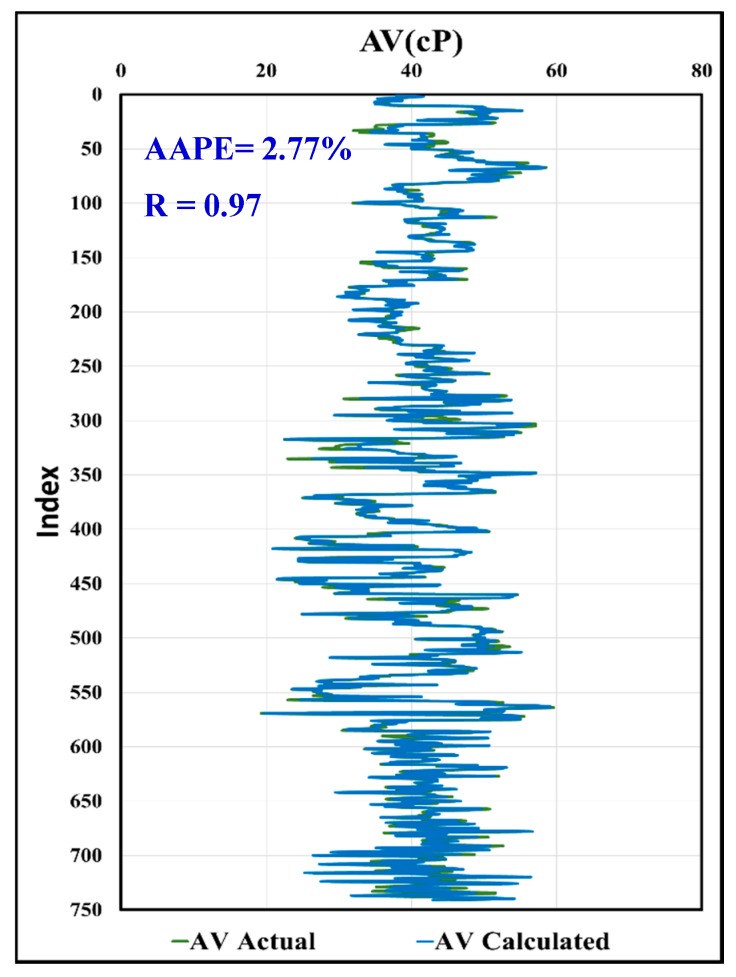
Actual AV vs. calculated AV using predicted R_600_.

**Figure 21:**
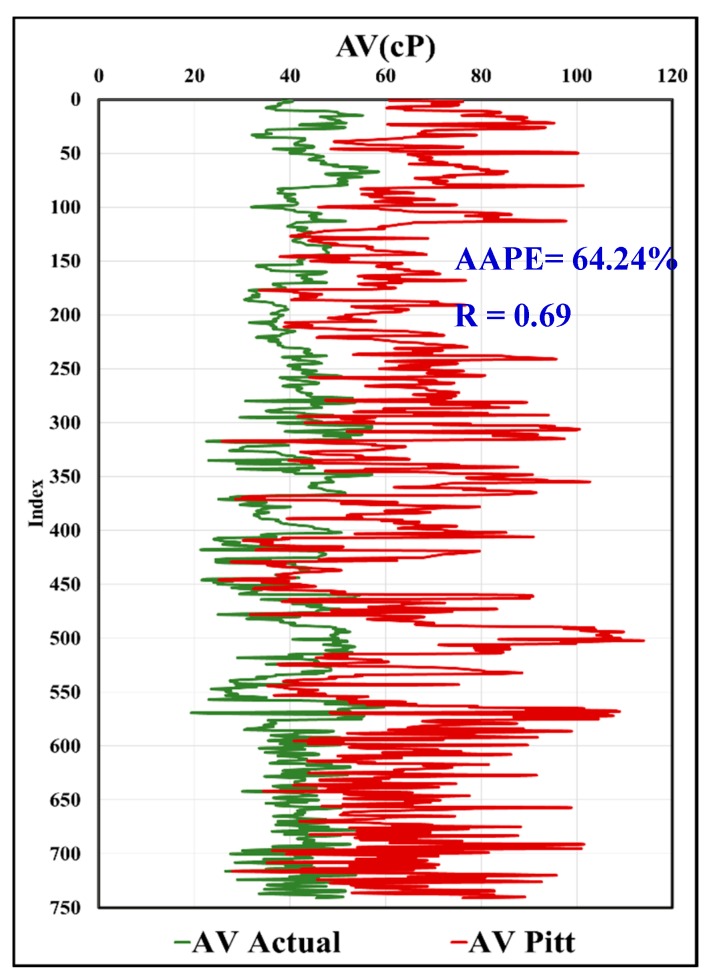
Actual AV vs. Pitt correlation.

**Figure 22:**
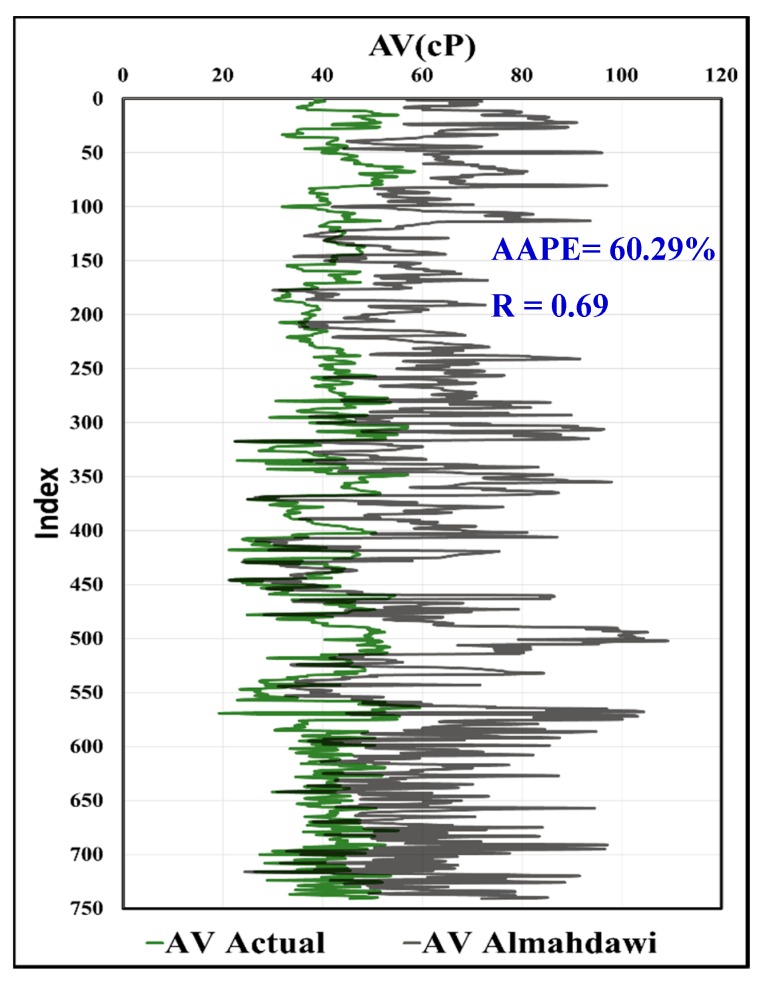
Actual AV vs. Almahdawi correlation.

In Figure 19, the predicted AV using the MWT and MFV was compared to actual AV with R equals to 0.97, and AAPE of only 2.77%. In Figure 20, the calculated values of AV from the predicted R600 using the MWT and MFV were compared to the actual values and showed the same result as the predicted AV, which is assuring the high accuracy of the model developed by this paper. This comparison included all the data used for training and testing. It shows the strength of the developed model using ANFIS. In addition, the same data (741 rows) of training and testing were used with correlations in the literature, which were proposed by Pitt and Almahdawi for calculating the AV from the same parameters (MWT and MFV) as in Equation (1) and Equation (2). Figure 21 shows the resulted values of AV from Pitt correlation as R is 0.69, and the AAPE is 64.24%. Figure 22 shows the resulted values of AV from Almahdawi correlation with AAPE of 60.29%, and R is 0.69. Table 3 summarizes all of these results. The results proved that the ANFIS models developed by this paper outperformed Pitt and Almahdawi correlations.

## 5. Conclusions

Adaptive neuro-fuzzy inference system (ANFIS) was used in this paper to construct the models to predict the invert emulsion mud rheological properties as PV, YP, R_600_, R_300_, behavior index, and AV using the more frequent field measurement MFV and mud weight as inputs. The work done before to utilize MFV in predicting rheological parameters has provided correlations that were including the solid percent. This paper is skipping the effect of solid percent in mud rheology using ANFIS technique in optimization to emulate the results of the training sets for different rheological properties. The developed models were tested and validated by separate testing sets, which were covering almost the same ranges of the training sets, which is assuring the quality of this work. The main point of strength of this work is that it provides the base for an expert computerized system that can be incorporated in an automated system containing sensors for measuring MWT and the time of Marsh funnel viscosity to have a real-time measurement of rheology with high accuracy. The MFV and MWT both have a strong relation with rheological properties of mud that can be exploited in developing correlations but, considering the classification of the data used as per the mud type. Data preprocessing and qualifying are considered to be the main challenges, and that might need a strategy that goes behind the engineering sense. 

The summary of the discussed results of this work can be summarized by the following points:

- ANFIS models were able to predict the rheological properties with high accuracy based on MWT and MFV only
- The ANFIS models predict the rheology of the invert emulsion mud system with an AAPE less than 4% and a correlation coefficient higher than 90% for all prediction models for the training and testing data sets.
- Using the predicted values for viscometer readings at 600 and 300 rpm to calculate the apparent and plastic viscosity is showing great accuracy for the calculated AV and PV.
- The ANFIS model outperformed the other AV correlations in the literature.
- The model results will help the drilling engineers to have better control over the hole cleaning parameters and monitor the drilling fluid rheology in real-time.

## Figures and Tables

**Table 1:** Data Descriptive Statistics.

	MWT (PCF)	MFV (s)	PV (cP)	YP (lb/100 ft^2^)	The Behavior Index, n	R_300_	R_600_	AV (cP)
**Minimum**	67.00	45.00	13.00	10.00	0.47	25.00	40.00	20.00
**Maximum**	98.00	98.00	47.00	31.00	0.80	72.00	119.00	59.50
**Mean**	82.26	73.48	30.00	22.68	0.65	52.63	82.63	41.31
**Standard Deviation**	6.80	11.05	6.31	3.28	0.05	8.31	14.36	7.18
**Kurtosis**	−0.86	−0.55	−0.24	1.14	0.42	0.14	−0.10	−0.10
**Skewness**	0.22	0.08	−0.07	−0.71	−0.33	−0.41	−0.26	−0.26

**Table 2:** A comparison between the variable ranges for training and testing.

Data Set		PV (cP)	YP (lb/100 ft^2^)	n	R_300_	R_600_	AV (cP)
Training Data	Minimum	13	10	0.47	25	40	20
	Maximum	47	31	0.80	72	119	59.50
Testing Data	Minimum	18	16	0.54	34	53	26.50
	Maximum	44	31	0.74	67	110	55

**Table 3:** Accuracy comparison between the ANFIS model and other literature correlations.

	AV (Pitt)	AV (Almahdawi)	AV Calculated from Predicted R_600_	AV Predicted
**AAPE%**	64.24	60.29	2.77	2.77
**R**	0.69	0.69	0.97	0.97

## Nomenclature

AV	Apparent viscosity (cP)
PV	Plastic viscosity (cP)
YP	Yield point (1b/100 ft^2^)
R_300_	Dial reading at 300 revolutions per minute (deg)
R_600_	Dial reading at 600 revolutions per minute (deg)
MFV	Marsh funnel time (s)
MWT	Mud weight (pcf)
AAPE	Average absolute percentage error
R	Correlation coefficient
n	Flow behavior index
cP	Centipoise
Pa.s	Pascal.second
rpm	Revolution Per Min
lb	Pound mass
AI	Artificial Intelligence

## Unit Conversion

cP	= 0.001 Pa.s
Lb/ 100 f^2^	= 0.4788 Pa
inch	= 2.54 cm
pcf	= 0.016 gm/cm^3^
